# Laser manufacturing of spatial resolution approaching quantum limit

**DOI:** 10.1038/s41377-023-01354-5

**Published:** 2024-01-02

**Authors:** Xiao-Jie Wang, Hong-Hua Fang, Zhen-Ze Li, Dan Wang, Hong-Bo Sun

**Affiliations:** 1 State Key Laboratory of Precision Measurement Technology and Instruments, Department of Precision Instrument, Tsinghua University, Beijing, 100084 China; 2 State Key Laboratory of Integrated Optoelectronics, College of Electronic Science and Engineering, Jilin University, 2699 Qianjin Street, Changchun, 130012 China

**Keywords:** Optical techniques, Laser material processing

## Abstract

Atomic and close-to-atom scale manufacturing is a promising avenue toward single-photon emitters, single-electron transistors, single-atom memory, and quantum-bit devices for future communication, computation, and sensing applications. Laser manufacturing is outstanding to this end for ease of beam manipulation, batch production, and no requirement for photomasks. It is, however, suffering from optical diffraction limits. Herein, we report a spatial resolution improved to the quantum limit by exploiting a threshold tracing and lock-in method, whereby the two-order gap between atomic point defect complexes and optical diffraction limit is surpassed, and a feature size of <5 nm is realized. The underlying physics is that the uncertainty of local atom thermal motion dominates electron excitation, rather than the power density slope of the incident laser. We show that the colour centre yield in hexagonal boron nitride is transformed from stochastic to deterministic, and the emission from individual sites becomes polychromatic to monochromatic. As a result, single colour centres in the regular array are deterministically created with a unity yield and high positional accuracy, serving as a step forward for integrated quantum technological applications.

## Introduction

Since early demonstrations of femtosecond laser as a three-dimensional (3D) processing tool^1–6^, microdevices with exciting optical, electronic, mechanical, and magnetic functions have been manufactured^7–13^, by which novel concepts from 3D quantum photonic integrated circuits to intelligent micro-robots are enabled^14–16^. Much effort in the past decade in this field has been devoted to improving manufacture spatial resolution, and several tens of nanometre feature sizes have been reported based on multiphoton absorption^1^, stimulation emission depletion^17,18^, far-field-induced near-field enhancement^19,20^, and photoexcitation-induced chemical bonding effects^21^. Nevertheless, advanced applications, such as single-electron transistors, single-photon emitters (SPE), single-atom memory, or quantum-bit devices, require higher manufacturing spatial resolution (<10 nm, far beyond the optical diffraction limit), for example, the ability to address single-atom defects complex (SADC) for initiating atom-like optical transitions.

Along this line, the direct laser writing technique has been recently implemented to generate point defects called colour centres in wide-bandgap materials, such as centres in diamond, silicon carbide, aluminum nitride, and hexagonal boron nitride (hBN)^22–30^. Colour centres in hBN, in particular, have gained prominence among quantum platforms due to the van der Waals layered crystal structure, making them easy for photonic integration^31,32^. However, current laser-written hBN colour centres suffer issues with stochastic yield and positional accuracy. In addition, there is a very high probability of forming multiple centres over individual sites, which is undesirable for many applications.

In this work, we propose and experimentally demonstrate close-to-atom scale manufacturing using a threshold tracking and lock-in (TTL) method, by which feature sizes are as small as a few nanometres, e.g., <5 nm, ~λ/100, approaching quantum uncertainty limit, are realised. It enables near unity yield fabrication of quantum emitter with high positional accuracy and minimal damage to the lattice, which persists the quality of optical properties of colour centres. We show the laser-induced colour centres exhibit high brightness, high emission purity, and high stability (no spectral diffusion and blinking). This close-to-atom scale laser manufacturing represents a significant step forward in scalable quantum photonic technologies.

## Results

### Threshold Tracking and Lock-in (TTL) technology for close-to-atom scale laser manufacturing

Material damages occur only if an irradiation laser is sufficiently strong, while the damage becomes invisible under optical imaging and not spectroscopically detectable due to the signal weakness when its size is reduced to atomic or close-to-atom scale. A primitive unit that is geometrically small but optically large enough to initiate unique absorption, emission, and scattering functions different from the background materials, for example, a monochromatic single-photon emitter, is here defined as a single-atom defect complex (SADC). Under the extreme case, a SADC consists solely of a single-atom defect like a vacancy, an interstitial atom, or a broken bond. Its size, therefore, ranges from an atom diameter to several nanometres. Prior demonstrations of laser writing in solids have been limited to several tens of nanometres (~λ/10)^33^. Now a question arises, is it possible to deterministically produce such a much smaller SADC by direct optical fabrication?

**Fig. 1:**
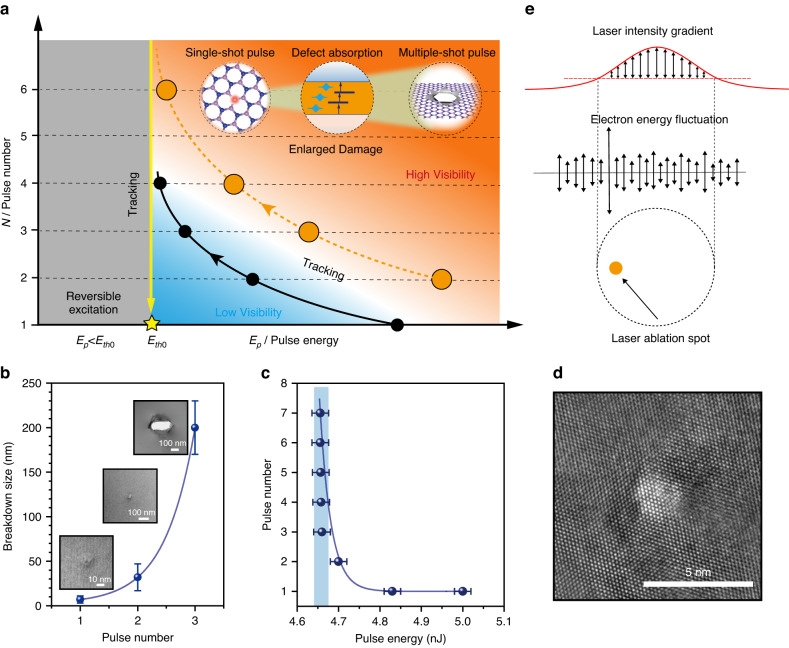
Creation of sub-5 nm SADC. **a** Schematic of the threshold tracking and lock-in technology. **b** Relationship between the damaging size on hBN versus pulse numbers. **c** Dependence of pulse number for producing optically visible defects on laser pulse energy **E**_**p**_. **d** TEM image of the laser-induced damaged region. **e** Schematic of laser ablated atom appears randomly in a small range defined by the statistical fluctuation of the electron kinetic energy when the laser pulse energy is reduced towards the SADC

To overcome this challenge, a technology called threshold tracking and lock-in (TTL) is proposed (Fig. 1a). The idea is to use the additional laser pulses as a probe to precise track the intrinsic threshold E_th0_ of crystal, which reflects the chemical bond strength of the material. By changing writing pulse numbers, we find that the laser-induced imperceptible damage to the lattice in hBN by the first pulse can be amplified by subsequent pulses, providing an opportunity for accurate determination of E_th0_. Traditionally, the damage can be characterised by irreversible modification in crystal observed in SEM or optical microscope. The crystal damage is more visible and easier to observe with higher laser power. However, the observations depend greatly on the sensitivity of the characterisation method, which means that experimentally defined thresholds E_thR_ can differ significantly for different experimental standards set. The TTL method, on the other hand, tracks the E_th0_ using the additional-laser-exposure dose, which is independent of the observation method. This was experimentally demonstrated in hBN flakes by changing the laser pulse number and energy. In Fig. 1b, the damaging area reduced from Φ ~ 200 nm to ~ 6 nm when the pulse energy was kept at E_p_ = 4.66 nJ while changing the shot number *N* from 3 to 1 (see more detailed data in Fig. S1 and Fig. S2). This fact enlightens us the experimentally defined E_thR_, is dependent on the experimental criteria, and the visibility of damaged area doesn’t reflect the intrinsic “threshold” for atomic defect formation. Figure 1c shows the dependence of shot number on pulse energy for the visibility of laser processed area under an optical microscope. 4.83 nJ is required for single-shot irradiation, while 2–5 pulses are needed for E_p_ = 4.70 to 4.66 nJ. A critical value, E_th0_ = 4.65$\pm$0.01 nJ, which is close to the lowest energy to produce a visible defect, is therefore deduced by the curve extrapolation when the material is processed with infinite laser shots. Irradiation with lower energy (<E_th0_) leads to reversible photoexcitation, and no permanent defects occur. With multiple shots amplification, the experimental determination of E_th0_ becomes independent either on observation methods (by imaging or by spectroscopy, optically or electronically) or on their sensitivity. Therefore, it is possible to track E_th0_, which is smaller than E_thR_ determined traditionally. More detailed experimental details are provided in Supplementary Note 1.

This E_th0_ is essential for close-to-atom scale manufacturing. Figure 1d exhibits the high-resolution transmission electronic (TEM) image of a SADC created by a femtosecond laser pulse. Although the morphology varies from spot to spot, the appearance of sub-5 nm feature sizes is highly reproducible (Fig. S3).

The advent of sub-5 nm SADC immediately evokes a question, what is the ultimate limit in femtosecond laser fabrication? Conventional laser damage threshold refers to a specific optical energy flux density, the critical energy absorbed per unit area that leads to excitation and collapse of lattice-electron subsystems^34^. In the view of statistical thermodynamics, the threshold is associated with a critical temperature of the subsystem that is scalable to a radius of a Gaussian beam, regardless of damaging mechanisms^35^. In this regard, the laser damaging area could be arbitrarily small until the single atom level if we lower the laser pulse energy sufficiently near the threshold. However, based on the continuous medium hypothesis, the model fails due to the statistic uncertainty limit. The energy of particles in a solid is distributed following probabilistic law (Fig. 1e). Single-atom ablation means that an atom gains sufficient kinetic energy to overcome the binding energy ${\epsilon }_{{\rm{b}}}$ and escapes from the system. The probability of the event occurrence, according to the Maxwell-Boltzmann distribution, is:

1 $$P\left(E\ge {\epsilon }_{b}\right)=1-{erf}\left(r\right)+\frac{2r}{\sqrt{\pi }}{e}^{-{r}^{2}}$$

where $r=\sqrt{\frac{{\epsilon }_{b}}{{k}_{B}T}}$ with ${k}_{B}$ being the Boltzmann constant, and $T$ is the temperature. When the laser energy E_p_ decreases towards the material damage threshold, the light intensity gradient at the Gaussian beam centre becomes less steep, the probability of single atom ablation is not geometrically truncated, which is affected by the statistic factor ${k}_{B}T$, that is, when $r\ll 1$, we have:

2 $$P\left(E\ge {\epsilon }_{b}\right)=\frac{2}{\sqrt{\pi }}\sqrt{{{\epsilon }_{b}/k}_{B}T}{e}^{-\frac{{\epsilon }_{b}}{{k}_{B}T}}$$

Following the analysis, laser ablation of a single atom doesn’t necessarily occur at the geometric centre of a laser focus when the laser pulse energy is reduced towards the SADC threshold but appears randomly in the small range, as shown in Fig. 1e. The uncertainly limit ${r}_{m}$, is estimated using$\,{\rm{\gamma }}=\varDelta {E}_{{kin}}/{U}_{p} \sim 1$, where $\varDelta {E}_{{kin}}$ is the statistical fluctuation of the electron kinetic energy, $\varDelta {E}_{{kin}} \sim {E}_{{kin}}/\sqrt{N}$, (*N* is the total number of excited electrons) and ${U}_{p}$ is the ponderomotive energy^36,37^. For hBN, we have ${r}_{m}$ ~ 3 nm (Supplementary Note 2), in accordance with the experimental observation of <5 nm.

### Close-to-atom scale manufacturing towards deterministic generation of single colour centres

**Fig. 2:**
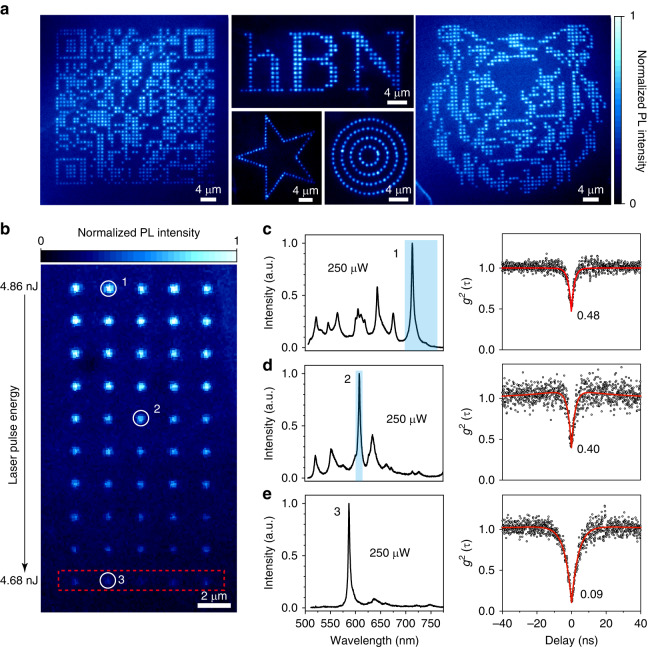
Deterministic creation of single-photon colour centres. **a** PL images of the colour centre patterns of quick response “QR” code, concentric circles, star, the letter “hBN” and “tiger” under the illumination of 450 nm light. The bright colour centres in these patterns were fabricated by single laser pulse with pulse energy *E*_*p*_ about (1 + 5%) E_*th0*_ ≈ 5.2 nJ. **b** PL image of hBN flakes after laser writing. The laser pulse energy decreases from the top to the bottom (from c, 4.86 nJ to e, 4.68 nJ). **c**, **d** Typical spectra of laser-written multiple SPEs and corresponding second-order autocorrelation measurement *g*^*2*^(*t*). The *g*^*2*^*(0)* value is marked for each curve. **e** Typical spectra of laser-written single-photon single-colour centres with low fluorescence background and its second-order autocorrelation measurement *g*^*2*^(*t*)

After demonstrating close-to-atom scale manufacturing, we now proceed to the colour centres generation in hBN flakes. One of the challenges in fabricating hBN quantum emitter is the low creation yield of active colour centres. We find that the close-to-atom scale manufacturing could yield a near deterministic generation of colour centres, as presented in Fig. 2a, which shows photoluminescence images of the colour centre pattern of quick response “QR” code, concentric circles, star, the letter “hBN” and “tiger” under the illumination of 450 nm light. Each site (fabricated by a single laser pulse with pulse energy E_p_ about (1 + 5%) E_th0_ ≈ 5.2 nJ) shows intensive emission, suggesting that they are all optically active, which could be created deterministically over each site.

Previous studies suggest that the single photon emission in hBN originates from the optical transition of energy levels from defect complexes, which are in the size of nanometres size or even smaller. Taking the donor-acceptor pair for example^38–40^, the wave functions of the electron on the donor and the hole on the acceptor overlap, then it can recombine and emit a photon. The typical radius of the donor-acceptor pair in hBN is around 10 nm^41^, which means that electrons and holes can recombine effectively within this range. Thus, single colour centres could be attained with a high probability if the size of laser processed site could be retained less than this range, which is achievable within our TTL method. With this in mind, we tested this concept experimentally by producing quantum emitter array with refined laser power. Figure 2b shows the fluorescence array generated by gently varied laser pulse energy. The emission spectra of the laser-written sites show a high correlation with the applied laser energy. Under high fluence, corresponding to large processed size, we observe ensemble emission possessing multiple emission peaks. As shown in Fig. 2c, more than ten peaks are discernible in the photoluminescence (PL) spectra from site 1, which is processed with a laser pulse energy of 4.86 nJ. The peak number of SADC could be reduced by finely reducing the laser energy. It drops to 4 for site 2 (Fig. 2d) and 1 for site 3 (Fig. 2e), when the sites are processed by laser energy of **E**_**P**_ = 4.78 nJ and 4.68 nJ, respectively. Site 3 shows a pure single-colour emission peak with the full width at half maximum of 3 nm. When the dot size shrinks to a close-atomic scale, here <5 nm for hBN, most of the sites exhibit only one sharp zero phonon line (Fig. 2e). This indicates that close-to-atom scale manufacturing could produce a single SPE with high yield, and the colour centre number over individual sites could be controlled to a certain extent.

To investigate the quantum nature of SADC, we perform photon autocorrelation measurements with the Hanbury Brown-Twiss (HBT) method. The experimental g^2^(t) data can be fit well with a three-level model: g^2^(t)$=1-a{e}^{-\frac{\left|t\right|}{{\tau }_{1}}}+b\,{e}^{-\frac{\left|t\right|}{{\tau }_{2}}}$ (Fig. S4). The antibunching curve at the isolated spectral window gives *g*^*2*^*(0)* value, an indicator of single-photon purity, as it is lowered from 0.48 (right, Fig. 2c) to 0.09 (right, Fig. 2e). Thus, it is reasonable to conclude that the multiple peaks in Fig. 2c and Fig. 2d come from a large population of colour centres within spatially unresolvable spots, emitting varied wavelengths. It is noted that these measurements were conducted without background correction. The *g*^*2*^*(0)* may be limited by several factors, including the single photon detector’s dark noise, the environment’s stray light, and residual organic fluorescence on the surface of hBN. Our result of the laser-induced single quantum emitter by near-threshold laser manufacturing is compared to other SPE fabrication methods such as nano-indentation, focused ion beams milling, and nanopillar substrates, which typically create SPEs with high background emission or cluster emitters within spatially unresolvable spots, resulting in reduced single photon purity.

### Highly bright, durable single photon emitter array

**Fig. 3:**
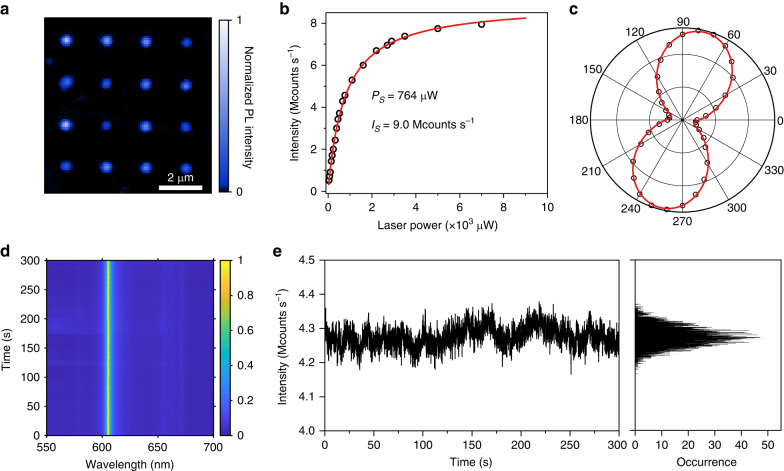
High reproducibility, high brightness, and high durability of single-photon single colour centres. **a** Fluorescence image of a 4 × 4 array of single-photon single colour centres induced by the same pulse energy. **b** Fluorescence saturation curve with a saturation count of 9 Mcounts s^-1^ with a saturation power of 764 μW. **c** Emission polarisation curves from the emitter. **d** The time series of PL spectra from the emitter. **e** Intensity time trace with a binning time of 50 ms acquired by APDs through the time-gated time-resolved method. The intensity histogram is plotted on the right

Then, we fabricate a single-photon emitter array with only one colour centre for each site (the single laser pulse energy is held at E_p_ = (1 + 0.5%) E_th0_). Figure 3a shows the confocal PL image of a dot array. The single-photon emitter array shows bright and durable single-photon emission. Fifteen of sixteen SADC (94%) are demonstrated to be single-colour SPE, which are identified with one ZPL (Fig. S5) and low photon correlations g^2^(0) (Fig. S6). In addition, among 150 laser-induced SPEs, 83% of emitters is below 0.3, 56% below 0.2, and 5% below 0.1 (Fig. S7). The emission brightness is tested by room-temperature PL intensity as a function of excitation laser power. Figure 3b shows a typical result of the sites. The fitting yields I_sat_ = 9.0 Mcounts s^-1^ with the saturation power P_sat_ = 764 μW. According to the statistics, ~91% of emitters exhibit single-photon count rates of >5 Mcounts s^−1^, and 20% of them exceed 10 Mcounts s^−1^ (For additional information see Supplementary Note 3, Fig. S8 and Fig. S9), which is higher than other reports^27,42^. Figure 3c shows the fluorescence polarisation measurements of this emitter. The curve is fitted by using a fitting function ${\cos }^{2}(\theta )$. The emission polarization visibility is calculated to be 77%, demonstrating the nature of single linearly polarized dipole transition.

Previous reports show that SPEs in hBN usually suffer spectral diffusion, blinking, and bleaching. To investigate their photostability, time-serial spectra of a single emitter were captured, as shown in Fig. 3d. The time-series spectra exhibit high spectral and intensity stability with negligible spectral diffusion or intensity fluctuations under one milliwatt continuous-wave laser excitation over 5 min. Moreover, Fig. 3e reports the intensity−time trace and corresponding intensity histogram acquired by avalanche photodiodes (APDs) through the time-tagged time-resolved (TTTR) method. The intensity−time trace with a binning time of 50 ms and histogram reflect that the emitter exhibits stable count rates of around 4.3 Mcounts/s with a fluctuation of 2.3% under the excitation of 1 mW, and no blinking or bleaching was observed (Fig. S10 and Fig. S11). We have tested 200 emitters, of which 188 emitters (94%) showed neglected wavelength diffusion and intensity fluctuation. In addition, the single emitter exhibits excellent stability under ambient conditions. We didn’t find emission intensity degradation after storage in the ambience for more than half a year (Fig. S12).

**Fig. 4:**
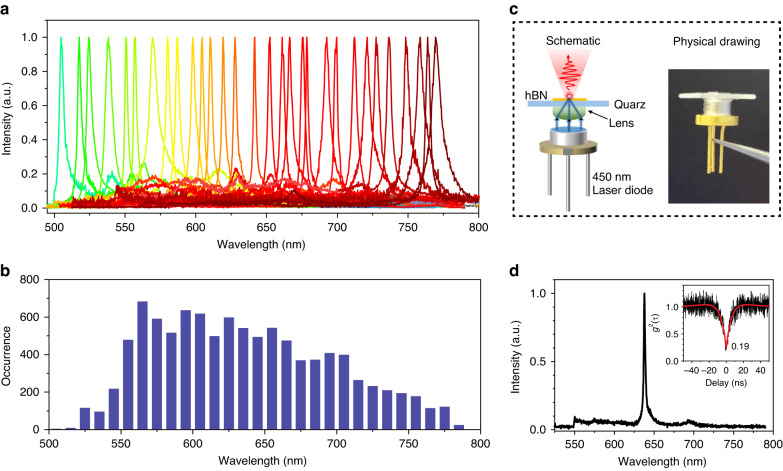
Monochromatic single-photon emitters from 500 nm to 800 nm. **a** The PL Spectra of laser-written single-photon single colour centres with emission ranging from 500 nm to 800 nm. **b** Histogram of ZPL wavelength distribution taken from more than ten thousand quantum emitters. **c** Schematic of the hybrid single-photon light-emitting device. The illustration shows the physical drawing of the device. **d** Representative laser-diode driven PL spectra of laser-written SPE. Inset is the antibunching curve of the corresponding spectra

Although monochromatic SPE could be obtained in the laser-processed site, the emission wavelength of SPE is still challenging to be controlled. Figure 4a shows the spectra of single photon emitters. In the experiment, the annealing process plays a vital role in restructuring and forming new optically active colour centres. Before annealing, no PL signal was observed from the laser-processed sample. After annealing, sharp, bright PL peaks at laser-written sites appear. High-temperature annealing could not only heal the lattice damage caused during the laser-writing process but also provide energy for the mobilisation of vacancies and self-interstitials, facilitating local bonding and activation of colour centres. A histogram of the spectral distribution of zero-phonon-line for approximately ten thousand centres indicates a vast distribution of emission colours (Fig. 4b). We noted that the occurrence of wavelength is mainly located in regions from 560 nm to 750 nm, while the probability for shorter or longer wavelength single-photon sources is much lower. The luminescent centres in our study have a wide range of spectral distribution, making it difficult to group them into previously identified defect types found in hBN. Earlier studies have shown the defect species in hBN like UV emitters, blue emitters, and visible emitters. However, the correlation between the PL spectrum and defect structure requires further investigation, which is beyond the scope of this paper. A further understanding of defect structures and the advancements in technology for controlling the doping of certain elements may increase the likelihood of obtaining a particular type of defect and reduce its wavelength distribution.

To fully utilise the broad range of colour centre emission wavelengths, SPEs of demanded colours are attained by packing a pre-selected flake with a 450 nm Laser diode (LD). Figure 4c illustrates an artistic view of the hybrid single-photon light-emitting devices developed in our work, comprising an hBN flake containing a single-photon emitter on top of a blue (450 nm) LD. Figure 4d shows representative LD-driven PL spectra of an emitter, which emits a peak at 636 nm under LD excitation. A second-order autocorrelation measurement on this emission site resulted in g^2^(0) = 0.19 seen in the inset. The hybrid single-photon light-emitting device shows the scalability of hBN quantum emitter in integrated applications. Additionally, the hybrid device could be integrated with optical fibers to create a more compact and portable product.

## Discussion

In summary, we report sub-5-nm close-to-atom scale precision femtosecond laser fabrication by threshold-tracking and lock-in technology, which is approaching the quantum uncertainty limit, a new milestone after the optical diffraction limit. The technology is employed for deterministically producing SADC in hBN. The SADC, recognised, in nature, as a single-colour centre, exhibits excellent performance of high purity, high brightness, and high durability. We also show that close-to-atom scale laser manufacturing enables single colour centre generation with an unprecedented high yield. This result suggests the high potential of close-to-atom scale laser manufacturing for the application of quantum devices.

## Materials and methods

### hBN sample preparation

We used hBN crystals (2D Semiconductors Inc., purchased from Sixcarbon Tech Shenzhen) to generate flakes using a Scotch-tape mechanical exfoliation method. The desired hBN flake was transferred onto a flat quartz substrate via a “dry transfer” technique based on a polydimethylsiloxane (PDMS) framework. The sample was then mounted on the three-dimensional stage for laser position and pattern.

### Laser writing setup

For close-to-atom scale laser manufacturing (Fig. S13), a commercial femtosecond laser (Pharos, light conversion) was used as a light source. The laser pulse was linearly polarized longitudinally along the plane, with a duration of about 230 fs and a wavelength of 1030 nm. The second harmonic generation was produced by using a BBO crystal. A high numerical aperture objective lens (NA = 0.95, 50 × Olympus) was used to tightly focus the laser pulse for fabricating colour centres. The measured full width at half maximum (FWHM) of the focal spot is ~357 nm (Fig. S14). A precision translation stage is used for the three-dimensional scanning. The pulse energy was controlled using a combination of a half-wave plate and a polariser and monitored by a photodetector before the objective lens.

### High-temperature annealing

High-temperature annealing was performed in a tube furnace following the laser-writing process. The samples were annealed at 1000 °C for 2 h at a 10^-4 ^Pa vacuum. Then, the samples were cooled down to room temperature for 4 h.

### Room-temperature optical characterisation

Optical measurement, including the confocal imaging, the fluorescence spectrum, and antibunching experiments, was based on our home-built confocal microscope, as shown in Fig. S15. A continuous-wave 488 nm laser was used for excitation. The laser was focused onto the sample using a high-numerical-aperture (NA = 0.95, Olympus) objective lens. The FWHM of the focal spot is 339 nm (Fig. S16). A polariser combined with a half-wave plate was used to control excitation power. For PL mapping and position, an X-Y-Z piezoelectric stage (PI instrument) was used. The collected fluorescence was filtered using a 500 nm dichroic mirror and an additional long-pass filter (Thorlabs FELH0500 or FELH0550). The signal was split by a beam splitter in the ratio of 30:70, and coupled into a grade-index fiber. One part of the signal was directed into a spectrometer (Princeton instruments) for collecting PL spectra, while the other part was directed into the two avalanche photodiodes (Excelitas, Dark count rate: around 80 Hz; Quantum efficiency: around 70% at 650 nm) for autocorrelation measurements. The fibre aperture serves as a confocal pinhole. Antibunching measurements were done using a time-correlated single-photon counting module (PicoHarp 300, PicoQuantum). The *g*^*2*^*(t)* data were not corrected for background luminescence.

### High-resolution TEM characterisation

High-resolution transmission electron microscopy (TEM) was conducted on JEOL (JEM-2100F) operating at 200 kV accelerating voltage.

### Supplementary information

Supplementary Information
