# Supplementary material for: Laser manufacturing of spatial resolution approaching quantum limit
Source: Light Sci Appl. 2024 Jan 2;13:6. doi: 10.1038/s41377-023-01354-5 (PMC10758390; doi:10.1038/s41377-023-01354-5)
Supplement: Supplementary file 1 — Supplementary Information [file 41377_2023_1354_MOESM1_ESM.docx]

**Supplementary Information for“Laser Manufacturing of Spatial Resolution Approaching Quantum Limit”**

Xiao-Jie Wang^1^, Hong-Hua Fang^1^*, Zhen-Ze Li^1^, Dan Wang^2^, Hong-Bo Sun^1,2^*

^1^ State Key Laboratory of Precision Measurement Technology and Instruments, Department of Precision Instrument, Tsinghua University, Beijing, 100084, China

^2^ State Key Laboratory of Integrated Optoelectronics, College of Electronic Science and Engineering, Jilin University, 2699 Qianjin Street, Changchun, 130012, China

*Corresponding author

Hong-Hua Fang^1^*, *hfang@mail.tsinghua.edu.cn;*

Hong-Bo Sun^1,2^*, *hbsun@tsinghua.edu.cn;*

**Supplementary Notes**

**Supplementary Note 1. Laser writing threshold tracking in hBN crystal**

Firstly, we perform the laser writing experiments as a function of the number of applied pulses and laser energy. The laser fabrication process of the sample is in situ monitored by a microscope (50× objective, NA 0.95) allowing for visualisation of its surface. Figure S1 shows a typical optical image of laser-produced matrices of 54 (6 × 9) in hBN taken by a CCD camera (Pixel size: 5.3 μm) in monochrome mode. The images are systematically normalised and binarized to avoid issues caused by different observer interpretations. The laser energy decreases laterally and the number of applied pulses increases longitudinally. We observe that the pulse number impacts the spot size significantly when the number is justly varied from one to three. From the optical image, the damage caused by the first pulse was not observable in the camera when laser energy was lower than 4.84 nJ. It became visible when the craters were produced with two pulses at the same laser energy. Three pulses are required when laser energy is lowered to 4.62 nJ. When the energy is below 4.60 nJ, no visible damage was observed, even with multiple pulses. In this method, we can determine the laser energy (E_th0_**)** for close-to-atom scale optical breakdown.

To further investigate the pulse number and laser energy effect on the size of the laser-produced spot, we repeated producing matrix and characterised finer features with TEM microscopy. Figure S2a shows a TEM image of the morphology of laser-induced modification in hBN flakes. From left to right, the pulse energy was increased from 4.66 nJ to 4.90 nJ. Each vertical axis of the grids was created with the same pulse energy but with varied pulse numbers from one to three, as marked in the image. As illustrated, the feature size is strongly dependent on the pulse numbers. A nanoscale lattice breakdown with a size of less than 10 nm (~ 6 nm) is induced by a single pulse with an energy of 4.66 nJ, as presented in higher magnification (Fig. S2b). The laser-induced size is increased to be more than 30 nm with two pulses, (Fig. S2d) and larger than 200 nm with three pulses. The sequent pulses irradiation will lead to an enlarged written void and induce uncontrolled cracks, which could be observed under an optical microscope. These findings enable us to push writing resolution to the physical limits of optical damage. The TEM image in Fig. S3 shows a typical laser-modified range with single pulse writing. We used the near-threshold energy E_p_ at 0.5% above E_th0_ to write the colour centre in hBN. The size distributes from 3~8 nm.

**Supplementary Note 2.** **The physical limit of determinism and precision**

- 1. **The traditional description of laser ablation under the continuous medium hypothesis**

In this section, we first review the physical description of the laser ablation process under the continuous medium hypothesis. In the typical experimental studies of the ultrafast laser-matter interaction,^1, 2^ the laser damage threshold is usually associated with the specific light energy flux $F_{th}$, as it determines the energy absorbed per unit area and ultimately leads to ultrafast heating of the electron/lattice system. One of the most famous equations describing this process is the two-temperature model^3^:

$C_{e}\frac{\partial T_{e}}{\partial t} = \nabla(k_{e}\nabla T_{e})-G_{el}(T_{e}-T_{l})+A$ (1)

$C_{l}\frac{\partial T_{l}}{\partial t} = G_{el}(T_{e}-T_{l})$ (2)

Where $C_{e}$ and $C_{l}$ in the equation represent the heat capacities of the electronic system and lattice system, respectively, $k_{e}$ is the thermal conductivity of the electron, and $G_{el}$ is the electro-acoustic coupling coefficient.

Although the mechanism of laser damage to materials can be divided into many types, such as coulomb explosion and thermal ablation/evaporation, these damage thresholds can be measured using a parameter critical temperature, $T_{cr}$, of a particular electron/lattice system. That is because when the system reaches a critical temperature, the total energy absorbed by the system exceeds the critical value of triggering a particular damage mechanism. Considering that Gaussian beam with *A ∝* $e^{-\beta r^{2}}$ is usually used in machining, the damaged area of the ideal laser machining should be at the geometric centre of the laser focus spot, and the radius of the damaged area will decrease rapidly as the laser energy gradually approaches the threshold $F_{th}$.

- 1. **The statistical (thermodynamics) description of laser-matter interaction**

The above theory implies that the radius of an area damaged by a laser can be continuously reduced to the level of a single atom. However, the assumptions underlying the above discussion will fail when it goes to the single-atom scale. This assertion is based on the following considerations: it becomes undefined when the laser energy is close to the so-called laser processing threshold. It is known from statistical physics that the relative average fluctuation grows up as the average number of microscopic particles goes to zero in an open system. The relative error in measuring the ablation threshold for a decreasing number of ablated atoms will increase. Therefore, the threshold for a single atom ablation is not a physically determinate value. In the case of the femtosecond laser-matter interaction, a laser-excited atom lattice remains intact, i.e., the macroscopic atomic motion could be neglected. An atom could be removed from a solid (ablated) if it receives energy above the binding energy, $\varepsilon_{b}$. It is possible to choose a low enough energy theoretically to obtain $P\left( E\geq\epsilon_{b} \right) \sim1/N_{0}$ to realise single-atom processing. However, according to statistical thermodynamics, the ablated atom must be randomly localised within the laser spot, even when the laser spot is spatially uniform. Thus, the position precision suffers an uncertainty limit. The uncertainty limit originates from the quiver energy acquired from the light field and thermodynamics. It can be described by using a minimum machining radius $r_{m}$, in which range the atomic ablation occurs, as discussed below.

In ultrafast laser ablation, the target material absorbs more photons than the minimum number required for photoionisation. The free carrier could obtain quiver kinetic energy from an oscillating electric field of the light field, according to:

$K\left( t \right)=K\left( t_{0} \right)-\frac{e}{\hbar}\int F(t')dt'$ (3)

where $K\left( t_{0} \right)$ is central crystal momentum, describing the carrier’s initial state; the second term represents the kinetic momentum of a free electron in an external electric field.

In the approximation of purely intraband motion, the electron's energy is periodically changed by the oscillating electric field. The cycled-average energy of intraband motion is called ponderomotive energy and can be described by the following equation:^4, 5^

$U^{n}\left( k \right)= \frac{1}{T_{0}}\int_{0}^{T_{0}} [E_{n}\left( K\left( t \right) \right)-E_{n}\left( k \right)]dt$ (4)

where *n* is the energy band index, $T_{0}$ is the oscillation period of the laser field with the main frequency ω. In the effective mass approximation, the ponderomotive energy of an electron-hole pair can be expressed as follows:

$U\left( k \right)=U= \frac{e^{2}E_{0}^{2}}{4\mu\omega_{0}^{2}}$ (5)

$\mu$ is the effective mass, *E_0_* is the amplitude of the oscillating electric field, and *ω_0_* is the frequency of the light field. Note that atomic ionisation processes are mainly produced near the maximum amplitude of the electric field. In the view of thermodynamics, kinetic energy can be described as temperature. The equipartition theorem tells us that the average kinetic energy is $E_{kin}=\frac{3k_{B}T}{2}$ for a carrier and the specific heat for the system from the laser is $C_{V}=\frac{3k_{B}N}{2}$. The fluctuation of the system temperature can be expressed as: ${\Delta E}_{kin}$=$\sqrt{\frac{2}{3N}}E_{kin}$, where *N* is the number of free carriers in the photoexcitation region. In the case of an ideal Gaussian beam focusing, the ponderomotive energy provides an initial gradient of the energy for ionization and defines the atomic ionization region spatially. However, when the laser intensity is reduced to a critical level close to the threshold, the number of excited electrons *N* is greatly reduced and mainly located at the spot centre. The kinetic energy fluctuations ${\Delta E}_{kin}$ of the system will be comparable with the additional Energy provided by Pondermotive motion $U$. When ${\Delta E}_{kin}/U$ ~ 1, we get the photon ablation uncertainty limit:

$r_{m}$ ~ $\sqrt{\frac{2}{3\pi\rho}}\frac{\overline{E}_{kin}}{U}$ (6)

where $\rho$ is the carrier density per unit area, and $\rho_{cr}=\epsilon_{0}\mu\omega^{2}/e^{2}$ is usually required for ultrafast laser ablation. For the single crystal of hBN, we consider $\rho$ ~ $0.1\rho_{cr}$, $\overline{E}_{kin}$~1 eV, and $\mu$ ~ 0.6. Therefore, we have $r_{m}$ ~ 3 nm, which is consistent with the experimental results.

**Supplementary Note 3.** **Photophysics of single photon emitters**

**3.1 *g^2^(t)* of the single-photon emitters**

Figure S4a and Fig. S6 show typical photon antibunching *g^2^(0)* statistics data obtained at room temperature. In addition, among 150 emitters, 83% of emitters are below 0.3, 56% below 0.2, and 5% below 0.1(Fig. S7). It is noted that these measurements were conducted without background correction. The *g^2^(0)* value may be affected by the dark noise of the single-photon detector and disturbances in the surrounding environment. The emission of a single photon in a solid-state defect system can be explained theoretically by a two-level model. However, these defect centres could be disturbed by the surrounding dielectric environment, which may introduce an intermediate level to participate in optical transitions. In our case, a three-level system was used to analyse the hBN quantum emitter, as shown in Fig. S4b.

The antibunching curves of these emitters were fitted by the following equation:

*g^2^(t)*$=1-ae^{-\frac{\left| t \right|}{\tau_{1}}}+b e^{-\frac{\left| t \right|}{\tau_{2}}}$

where parameters $\tau_{1}$ and $\tau_{2}$ are lifetimes of the excited and meta-stable states, respectively. We performed statistics of the $\tau_{1}$ and $\tau_{2}$ extracted by a fitted curve. The $\tau_{1}$ value is 1~5 ns, while $\tau_{2}$ value is 10~50 ns.

**3.2 Statistics of the distributions of saturation intensity**

To evaluate the brightness of laser-created quantum emitters, we recorded their PL intensity as a function of incident laser power and fitted experimental data using a model: *I=I*_sat_ *P/(P+P*_sat_*)*, where *I (I*_sat_*)* and *P (P*_sat_*)* are the PL intensity (saturation intensity) and laser power (saturation power), respectively. The representative data and fitting curves are shown in Fig. S8. Figure. S9a shows statistics of the distributions of saturation intensity with respect to emission wavelength. The saturation intensity of more than 80% of emitters exceeds 5 Mcounts s^-1^ and nearly 15% exceeds 10 Mcounts s^-1^, as shown in Fig. S9b. A wide range of emitter intensities are observed in our experiments. This may arise from different light absorption capacities or radiation modes, including non-radiative means to certain lower energy radiation states or other defects.

**3.3 Stability of an individual single-photon emitter**

Another key property of emitters is their optical stability. Figure. S10 shows the time-series spectra mapping over 5 minutes for part of the emitters. No spectral diffusion or intensity fluctuation was observed. We have tested 200 emitters, of which 188 emitters (94%) showed neglected wavelength diffusion and intensity fluctuation. We also record the time trace of intensity with a binning time of 50 ms acquired by APD, during which no blinking or bleaching occurred, as shown in Fig. S11. In addition, the single emitters exhibit nearly perfect stability under ambient conditions. We didn’t find emission intensity degradation after storage on the shelf for more than half a year (Fig. S12). These results show the superiority of close-atom-scale manufacturing by single laser pulse for inducing spectral-stable single photon emitters.

**Supplementary Figures**

**
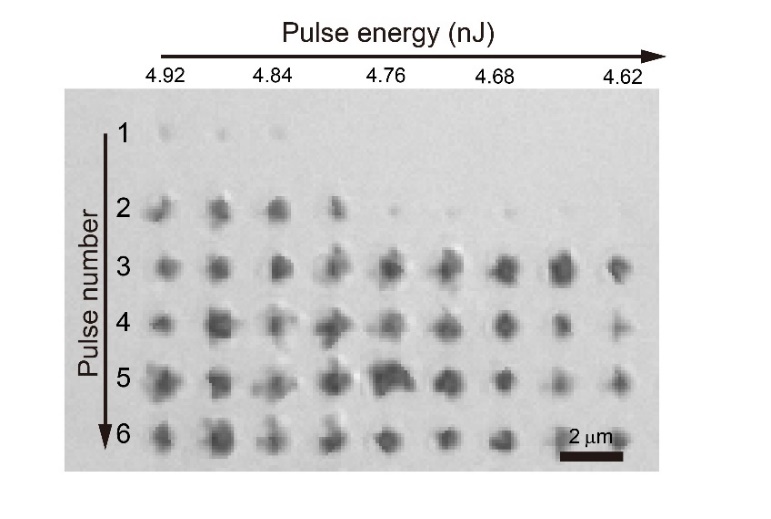
**

Figure S1. Optical image of laser-written hBN flakes **processed with varying pulse energy and pulse number.** The number of pulses and the pulse energy are marked in the image. (The thickness of the hBN flake is ~70 nm)


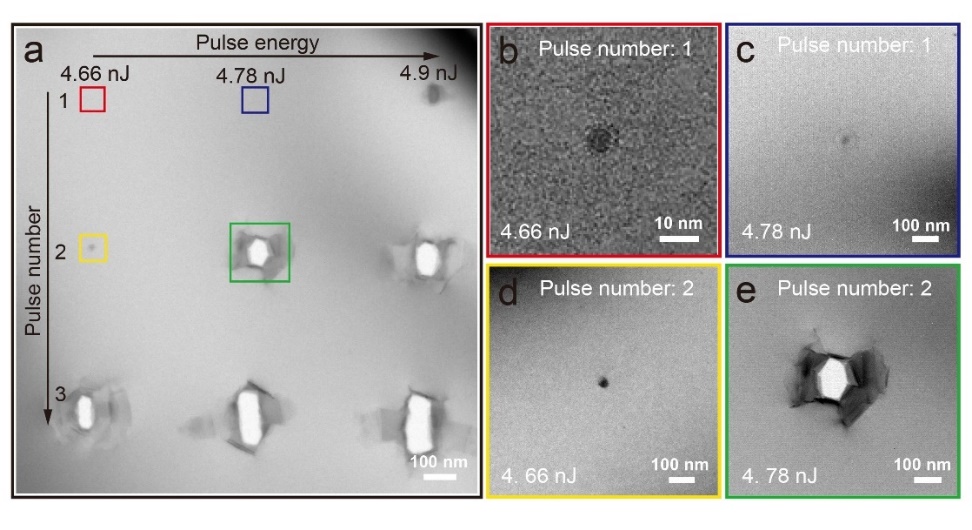


Figure S2. TEM images of laser-induced lattice modification. a The number of pulses and the pulse energy increase longitudinally and laterally, respectively, as marked in the image. **b, c** TEM images of single-pulse induced hBN morphology with pulse energy of 4.66 nJ and 4.78 nJ, respectively; **d, e** TEM images of two-pulses induced hBN morphology with pulse energy of 4.66 nJ and 4.78 nJ, respectively.

**
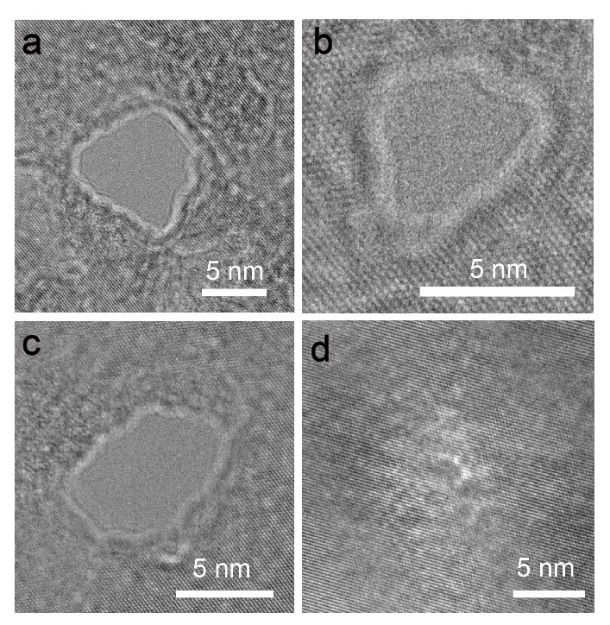
**

Figure S3. **TEM images of laser-induced nanoscale damage in different hBN flakes.** (Electron beam scan during SEM or TEM can affect the size of the measured ablated hole^6^.)


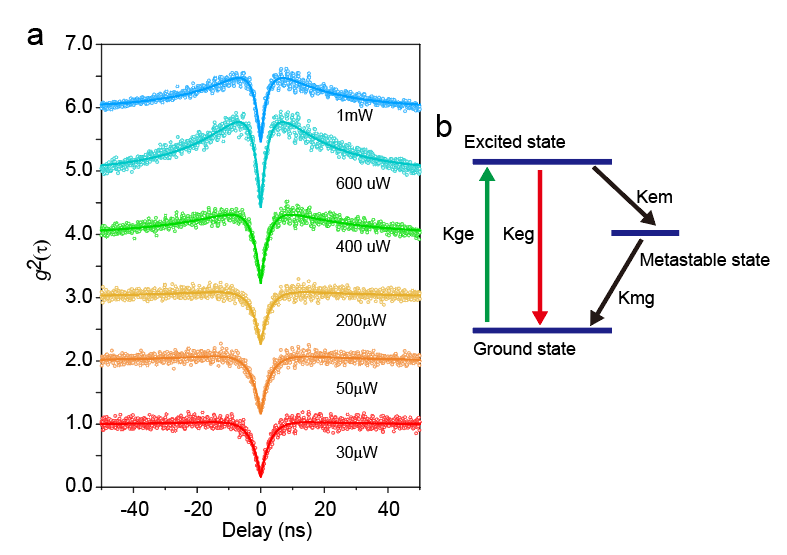


Figure S4. Second-order correlation functions as a function of pump power. **a,** Second-order autocorrelation function obtained different laser power. The pump power goes from 30 μW to 1000 μW. For the sake of visibility, the graphs are shown with an offset; **b,** The autocorrelation functions at high excitation power exhibit the typical shape of a three-level single photon emitter with the presence of a long-lived metastable state. A schematic diagram of a three-level model is used to describe the transition states of the emitters.

**
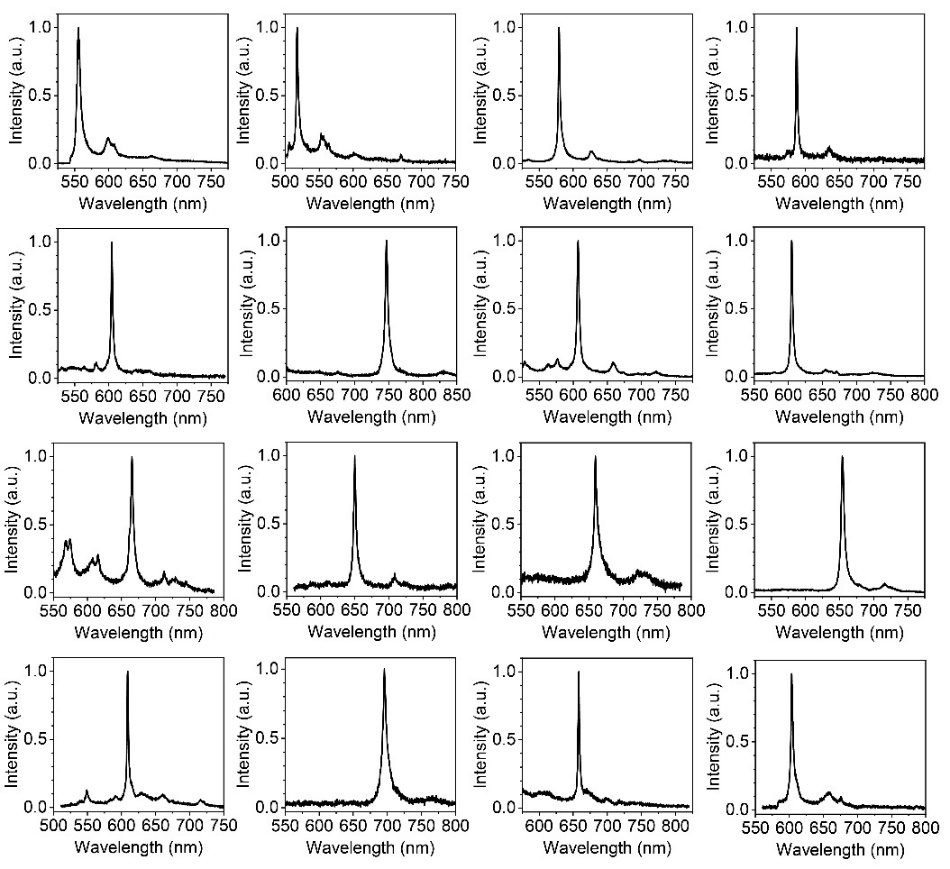
**

Figure S5. The spectra of laser-written SPEs pattern in Figure 3a. Each curve was acquired for 2s using a 250 μW, 488 nm CW laser as the excitation source.

**
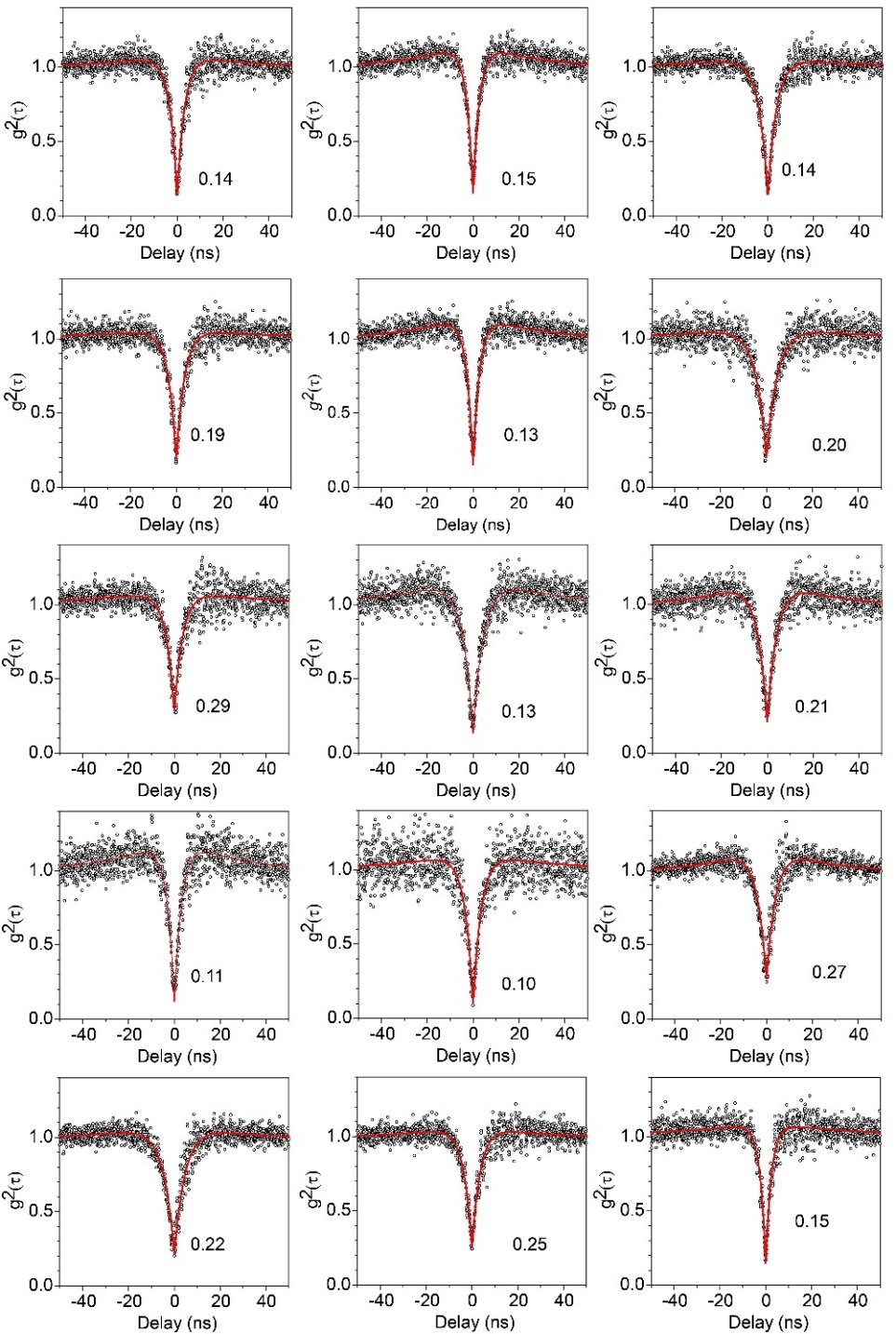
**

Figure S6. Antibunching curves from laser-written QEs. The *g^2^(0)* value is marked for each emitter. Each curve was acquired for 2 min using a 100 μW, 488 nm CW laser as the excitation source.


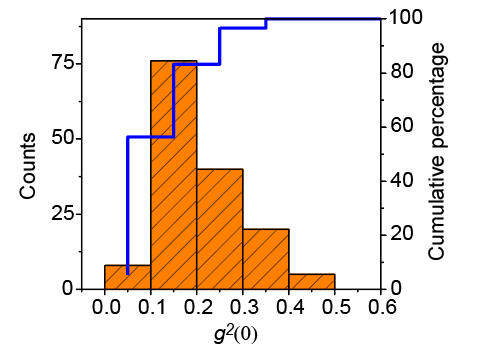


Figure S7. **Histogram of the *g^2^(0)* value of nearly 150 emitters.** Among 150 emitters, 83% of emitters are below 0.3, 56% below 0.2, and 5% below 0.1. It is noted that these measurements were conducted without background correction.


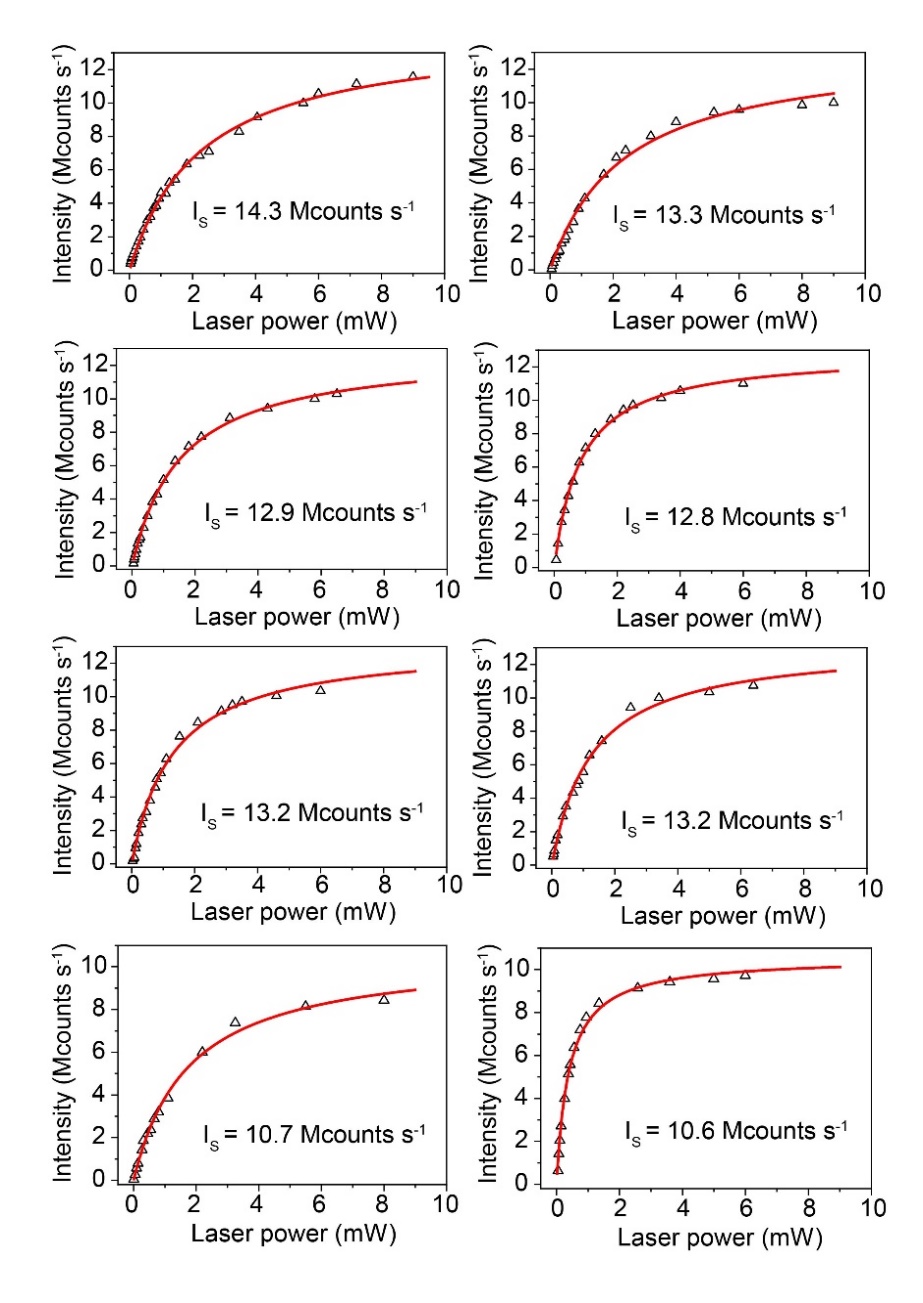


Figure S8. Fluorescence saturation curve of typical SPEs. The saturation intensity has been marked for each curve.


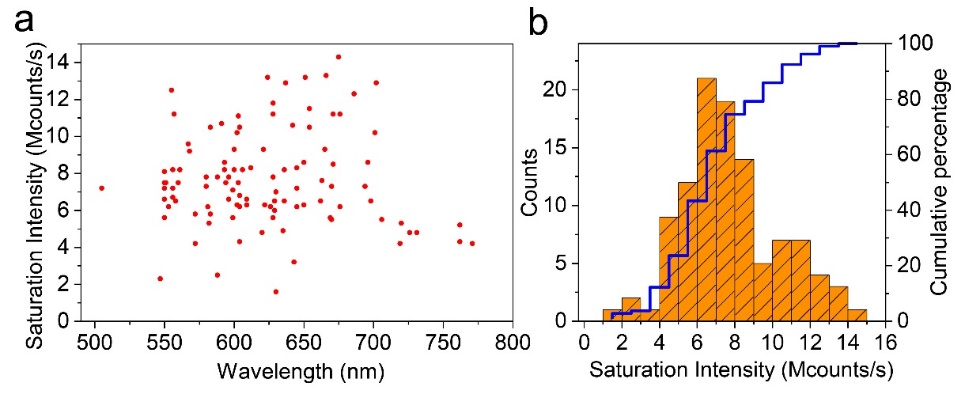


Figure S9. Statistics of the distributions of saturation intensity of above 100 emitters. **a** Distribution of ZPL vs saturation intensity from laser written quantum emitters; **b** Histogram of saturation intensity and cumulative percentage. The saturation intensity of more than 80% of emitters exceeds 5 Mcounts s^-1^ and nearly 15% exceeds 10 Mcounts s^-1^.


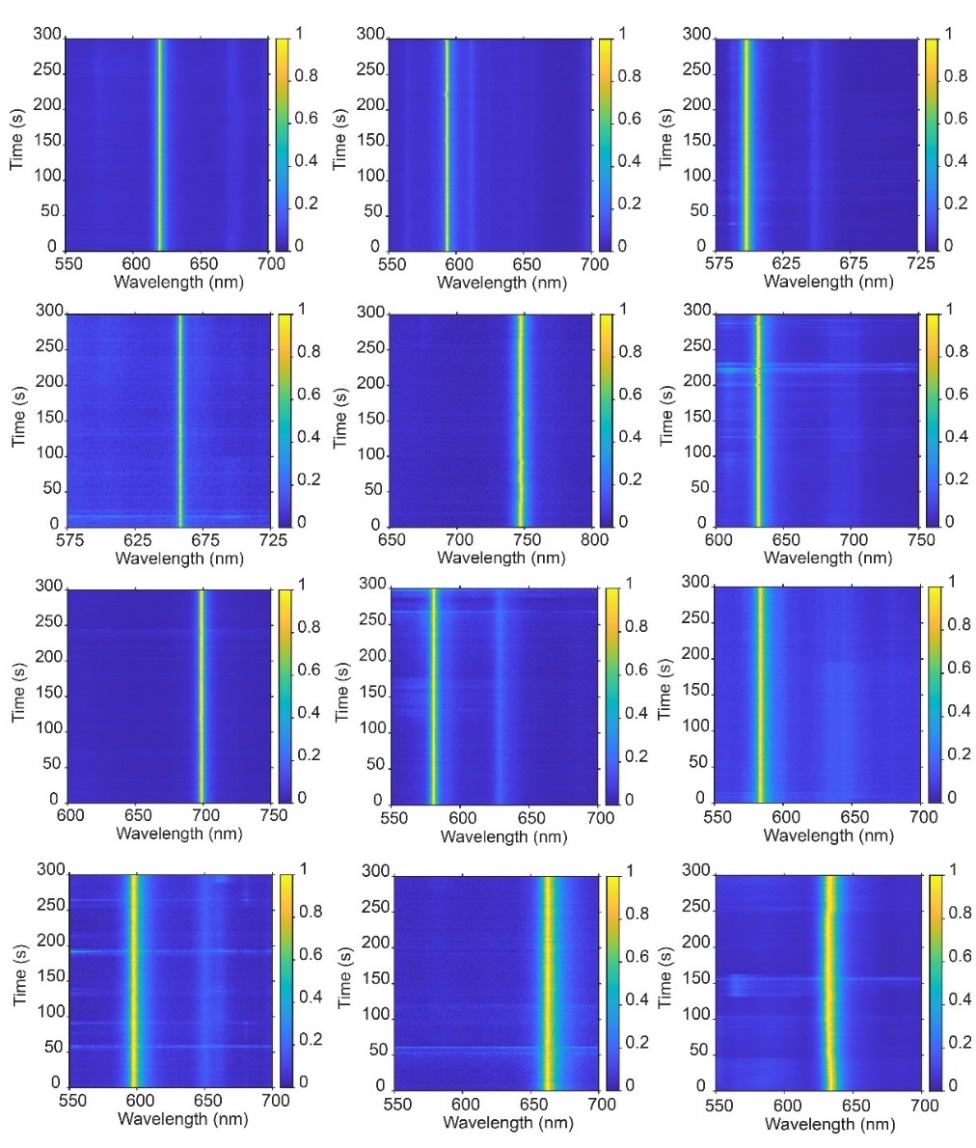


Figure S10. Room-temperature PL time series taken from laser-written SPEs. A frame is captured every two seconds. The time-series spectra exhibit high spectral stability with negligible spectral diffusion or intensity fluctuations under one milliwatt continuous-wave laser excitation over 5 minutes


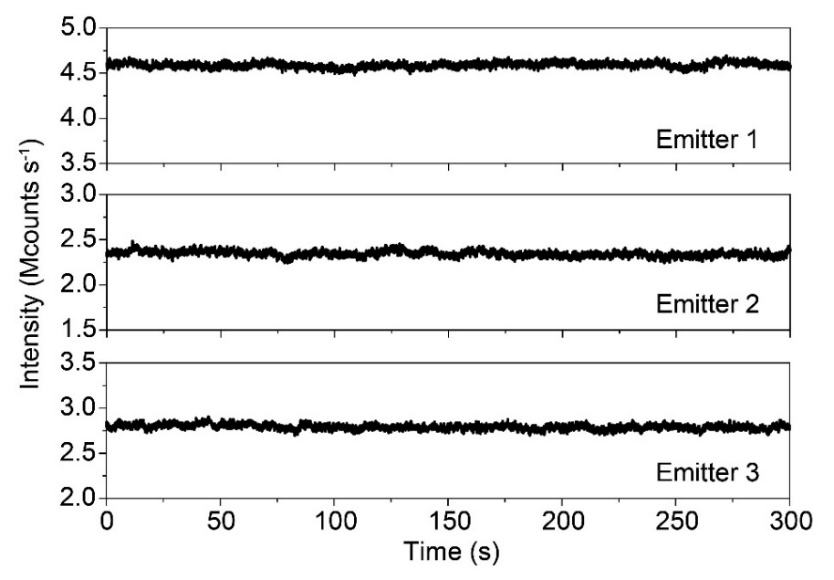


Figure S11. Intensity time trace acquired by APDs through the time tagged time-resolved method. The intensity−time trace with a binning time of 50 ms reflects that these emitters exhibit stable count rates under the excitation of 1 mW, no blinking or bleaching was observed.


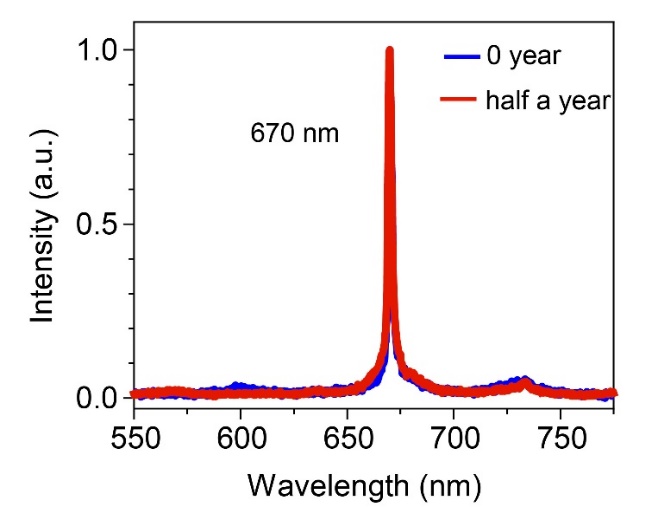


Figure S12. PL spectra of a representative emitter for half a year**.** Each curve was acquired for 2s using a 300 μW, 488 nm CW laser as the excitation source.


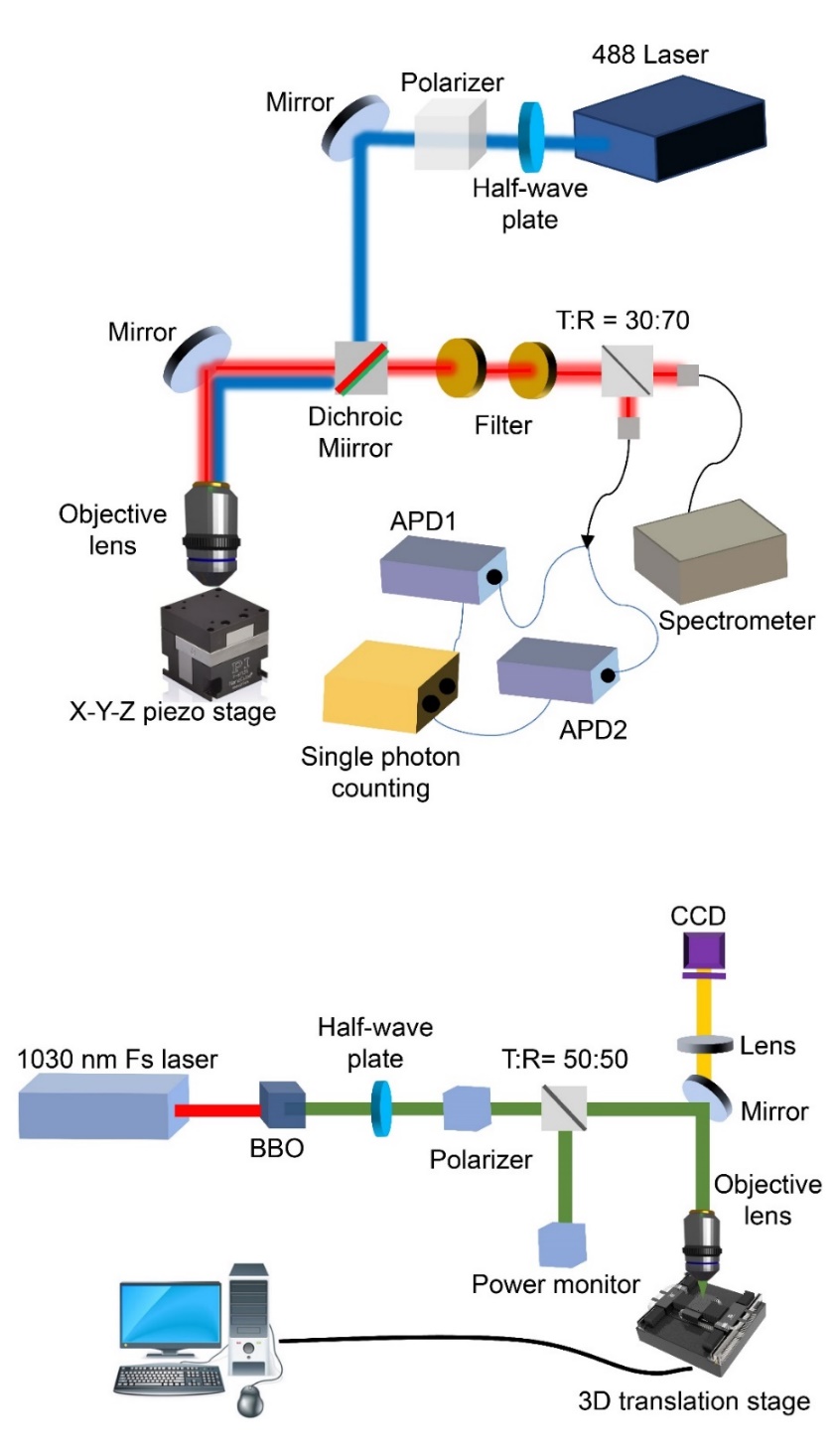


Figure S13. Schematic of the setup for ultrafast laser direct writing. A Yb:KGW amplifier at a wavelength of 1030 nm is used as the light source. 515 nm laser is produced by the second-harmonic generation of 1030 nm using (beta-BaB_2_O_4_) crystal. Pulse energy is controlled using a combination of a half-wave plate and a polariser. The beam splitter (50/50 splitting ratio) is inserted, and the laser pulse is monitored by a photodetector.

**
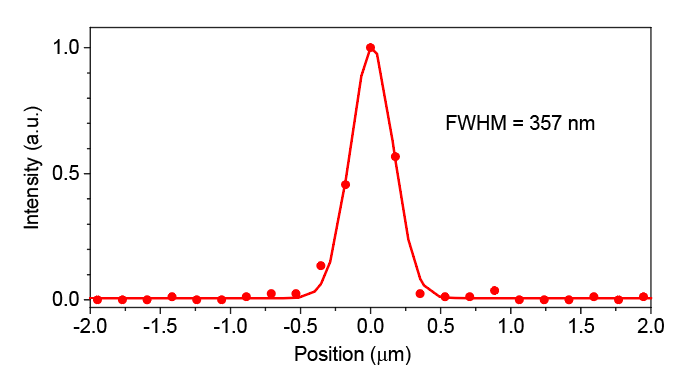
**

Figure S14. The size fitting of the focal spot of the 515 nm femtosecond laser. The circle and the solid traces are the experimental data and a Gaussian fit, respectively.


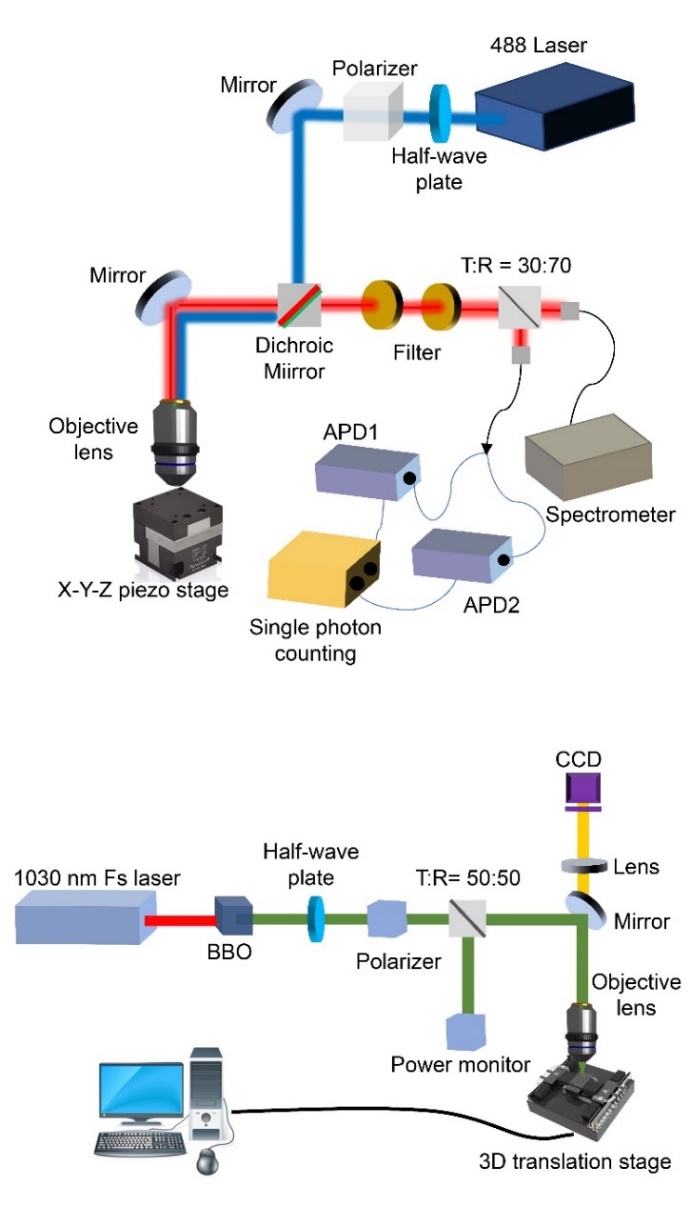


Figure S15. **Optical measurement confocal microscopy setup.** In the excitation path, a continuous wave (CW) 488 nm laser is focused on the sample after passing through the image relay system. In the detection path, the fluorescence photons are routed through a beam splitter toward the APDs or the spectrometer.

**
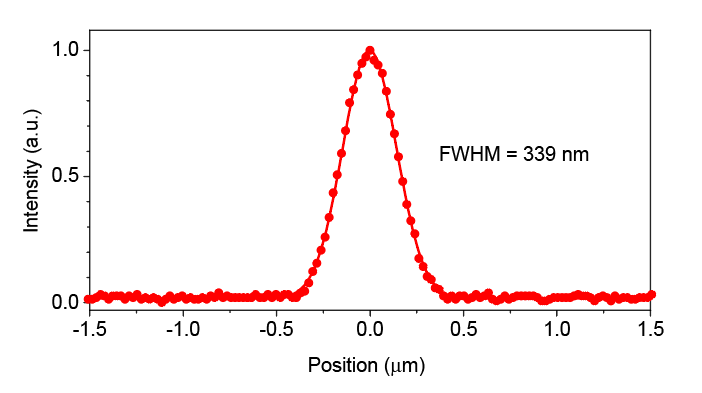
**

Figure S16. The size fitting of the focal spot of the 488 nm CW excitation laser. The circle and the solid traces are the experimental data and a Gaussian fit, respectively.

**Supplementary References**

1. Sudrie, L., A. Couairon, M. Franco, et al. Femtosecond Laser-Induced Damage and Filamentary Propagation in Fused Silica. *Phys. Rev. Lett.* 89, 186601 (2002).

2. Song, J., X. Wang, X. Hu, et al. Formation mechanism of self-organized voids in dielectrics induced by tightly focused femtosecond laser pulses. *Appl. Phys. Lett.* 92, 092904 (2008).

3. Christensen, B. H., K. Vestentoft and P. Balling. Short-pulse ablation rates and the two-temperature model. *Appl. Surf. Sci.* 253, 6347-6352 (2007).

4. Dombi, P., Z. Pápa, J. Vogelsang, et al. Strong-field nano-optics. *Reviews of Modern Physics.* 92, 025003 (2020).

5. Kruchinin, S. Y., F. Krausz and V. S. Yakovlev. Colloquium: Strong-field phenomena in periodic systems. *Reviews of Modern Physics.* 90, 021002 (2018).

6. Seniutinas, G., et al. Tipping solutions: emerging 3D nano-fabrication/ imaging technologies. 6, 923-941 (2017).
